# Identification and Characterization of Microsporidia from Fecal Samples of HIV-Positive Patients from Lagos, Nigeria

**DOI:** 10.1371/journal.pone.0035239

**Published:** 2012-04-09

**Authors:** Oladele Teslim Ojuromi, Fernando Izquierdo, Soledad Fenoy, Adetayo Fagbenro-Beyioku, Wellington Oyibo, Alani Akanmu, Nkiruka Odunukwe, Nuno Henriques-Gil, Carmen del Aguila

**Affiliations:** 1 Department of Zoology, Faculty of Science, Lagos State University, Ojo, Lagos, Nigeria; 2 Facultad de Farmacia, Universidad CEU San Pablo, Madrid, Spain; 3 Department of Medical Microbiology and Parasitology, College of Medicine, University of Lagos, Lagos, Nigeria; 4 Department of Hematology, College of Medicine, University of Lagos, Lagos, Nigeria; 5 Clinical Research Division, Nigerian Institute of Medical Research, Lagos, Nigeria; Tulane University School of Public Health and Tropical Medicine, United States of America

## Abstract

**Background:**

Microsporidia are obligate intracellular parasites that infect a broad range of vertebrates and invertebrates. They have been increasingly recognized as human pathogens in AIDS patients, mainly associated with a life-threatening chronic diarrhea and systemic disease. However, to date the global epidemiology of human microsporidiosis is poorly understood, and recent data suggest that the incidence of these pathogens is much higher than previously reported and may represent a neglected etiological agent of more common diseases indeed in immunocompetent individuals. To contribute to the knowledge of microsporidia molecular epidemiology in HIV-positive patients in Nigeria, the authors tested stool samples proceeding from patients with and without diarrhea.

**Methodology/Principal Findings:**

Stool samples from 193 HIV-positive patients with and without diarrhea (67 and 126 respectively) from Lagos (Nigeria) were investigated for the presence of microsporidia and *Cryptosporidium* using Weber’s Chromotrope-based stain, Kinyoun stain, IFAT and PCR. The Weber stain showed 45 fecal samples (23.3%) with characteristic microsporidia spores, and a significant association of microsporidia with diarrhea was observed (O.R.  = 18.2; CI: 95%). A similar result was obtained using Kinyoun stain, showing 44 (31,8%) positive samples with structures morphologically compatible with *Cryptosporidium* sp, 14 (31.8%) of them with infection mixed with microsporidia. The characterization of microsporidia species by IFAT and PCR allowed identification of *Enterocytozoon bieneusi*, *Encephalitozoon intestinalis* and *E. cuniculi* in 5, 2 and 1 samples respectively. The partial sequencing of the ITS region of the rRNA genes showed that the three isolates of *E.bieneusi* studied are included in Group I, one of which bears the genotype B.

**Conclusions/Significance:**

To our knowledge, this is the first report of microsporidia characterization in fecal samples from HIV-positive patients from Lagos, Nigeria. These results focus attention on the need to include microsporidial diagnosis in the management of HIV/AIDS infection in Nigeria, at the very least when other more common pathogens have not been detected.

## Introduction

Diarrhea and severe weight loss are syndromes described in HIV patients and known as “slim disease” by sub-Saharan Africans [1]. This pathology can be produced by several organisms, and it is commonly associated with *Cryptosporidium*. Additionally, microsporidia are considered as emerging pathogens worldwide and several species are involved in human disease [2]. Among them, *Enterocytozoon bieneusi* and *Encephalitozoon* species, *E. intestinalis*, *E. hellem* and *E. cuniculi* are the most frequently identified microsporidia in human clinical samples. They have been described as opportunistic pathogens in human immunodeficiency virus (HIV)-infected patients and other immunocompromised patients such as transplant recipients [3], [4], [5], [6], [7], [8], [9], [10]. However, microsporidia have also been detected in HIV-negative individuals [11], [12], [13], [14], [15] and it has been recently suggested that the incidence of miscrosporidial infections in healthy population is much higher than previously reported and microsporidia may represent neglected etiological agents of more common diseases [16] In HIV-infected patients, especially those with CD4+ T-cell counts below 100 cells per mm^3^, *E. bieneusi* and *E. intestinalis* have been associated with acute and chronic diarrhea [13], [17], [18], [19], [20], [21], [22].

In the last decade, epidemiological studies of human microsporidiosis, have been strengthened with the improvement of diagnostic methods and the development of molecular markers [23]. However, the transmission and sources of infection are not well understood. The transmission may involve person-to-person as well as waterborne or food contamination, especially in developing countries with poor sanitation [24]. In the last few years several authors have focused on animal microsporidiosis, in order to elucidate the possible zoonotic origin of human microsporidiosis. In fact, human microsporidia species have been isolated from a large number of domestic and wild animals [25], [26], [27], [28]. This zoonotic transmission is supported by phylogenetic studies which demonstrate that several genotypes can infect both humans and animals [29], [30], [31], [32].

The introduction of highly active antiretroviral therapy (HAART) with protease inhibitors to treat HIV/AIDS patients have substantially decreased the incidence of microsporidiosis in Europe [33]. However the situation differs in developing countries, where the rapid expansion of AIDS together with limited access to HAART has contributed to an increased incidence of this disease. In the studies carried out in Africa to evaluate the prevalence of microsporidiosis in HIV–infected patients due to *E. bieneusi* and *Encephalitozoon* species, a variable incidence ranging from 7 to 51% was obtained, depending on the population studied and the diagnostic methods used [11], [34], [35], [36], [37], [38], [39], [40], [41]. Unfortunately, epidemiological studies of microsporidiosis based in genotyping methods are scarce in these countries [42], [43], [44], [45], [46], so there is still insufficient information on the magnitude of the microsporidial infection in African countries. The most likely explanations are the lack of an easy to make diagnostic method specific for the detection of the parasite and the lack of experienced and skilled microscopists able to detect the organism in biological samples.

With the aim of contributing to the knowledge of the molecular epidemiology of microsporidia in Nigeria, we performed a study in HIV-infected patients with and without diarrhea. Additionally, and simultaneously, the presence of *Cryptosporidium* in these patients was also determined.

## Materials and Methods

### Ethics Statements

The protocol and studies were approved by the Institutional Review Board of the College of Medicine of the University of Lagos, Nigeria (CMUL) and the Nigerian Institute of Medical Research (NIMR), Yaba, Lagos, Nigeria. All participants gave informed consent before their samples were collected and processed.

### Study Population

A total of 193 patients were enrolled in the study, all of them were HIV–infected patients attending to referral clinics in Lagos, Nigeria, 67 (34.7%) with and 126 (65.3%) without diarrhea. Chronic diarrhea was either continuous, defined by the Center for Disease Control and Prevention as two or more loose stools per day for more than 28 days; or acute, defined as episodes of two or more loose stools per day alternating with episodes of formed stools for less than 28 days. Complete medical histories were obtained from the hospital records (Demographic and Clinical) for patients included in the study.

### Stool Sample Collection, Storage and Staining Methods

Fresh stool samples were collected in clean universal bottles labeled with each patient’s details. Samples were stored at -80°C until use. Thin smears were made from all fecal samples to be stained with Webeŕs Cromothrope stain and Kinyoun stain to detect the presence of microsporidia and tentatively *Cryptosporidium sp* [47], [48].

### Indirect Immunofluorescence Antibody Test (IFAT)

This was performed on all samples which tested positive by Webeŕs Cromothrope stain. IFAT was performed by coating the slides with 5 µl of each unconcentrated stool sample. Specific polyclonal antibodies from rabbit for *E. hellem* (1/500), *E. intestinalis* and *E. cuniculi* (1/400) [49], [50], [51] and a monoclonal antibody for *E. bieneusi* [52] were used. Subsequently, the fluorescein isothiocyanate-conjugated anti-rabbit (Sigma Cat. F-9887) and anti-mouse (Sigma Cat. F-1010) were appropriately diluted and the slides were examined with a fluorescence microscope.

### Molecular Analyses

#### DNA isolation

DNA from fecal samples found to be positive for microsporidia by the staining method was extracted by bead disruption of spores using the Fast-DNA-Spin kit, according to the manufactureŕs instructions (Bio 101, Carlsbad, Calif.). PCR inhibitors were removed using the QIAquick PCR kit (QIAGEN, Chatsworth, CA).

#### Species characterization by PCR

Microsporidial small subunit rRNA (SSU-rRNA) coding regions were amplified using the following species-specific primers EBIEF1/EBIER1 for *E. bieneusi* [53], SINTF/SINTR for *E. intestinalis* [54], ECUNF/ECUNR for *E. cuniculi* and EHELF/EHELR for *E. hellem* [55]. PCR amplification was performed with the GeneAmp kit (Perkin-Helmer Cetus, Norwalk, CT) according to the manufacturer instructions. The conditions for the reaction and the testing of purified samples for the presence of PCR inhibitors by spiking the samples with the corresponding cloned SSU-rRNA coding region was as described [56].

#### Genotyping by DNA sequencing analysis

*E. bieneusi* genotypes from 3 fecal samples were analyzed by nucleotide sequencing of the Internal Transcribed Spacer (ITS) of the rRNA genes. The PCR products were purified using Concerted Rapid PCR kit (GIBCO-BRL) and sequenced in both directions using an ABI Big Dye Terminator kit (v1.1) and an ABI 3100 automated sequencer (Applied Biosysytems). The sequences were edited with BIOEDIT 7.0 program [57] and aligned following the Clustal W algorithm [58].

#### Phylogenetic analyses

These were performed by comparison with 179 sequences available in the GenBank obtained from different hosts and reviewed by Henriques-Gil et al 2010 [59]. The haplotypes were generated with the program DNASP 4.10 [60], without considering the occasional gaps. Haplotype Median Joining networks [61] were performed with NETWORK 4.5.1.0 program (Fluxus Technology Ltd.). The network was simplified reducing star-like clusters to the ancestral haplotypes with a single run of star contraction [62].

### Statistical Analyses

The chi-square test (SPSS 15.0) was used to demonstrate the association between diarrhea and microsporidia or *Cryptosporidium* identification in fecal samples, and the correspondent odds ratio was calculated.

## Results

### Staining Methods

Forty-five stool samples (23.3%) showed a variable number of spores that stained pinkish red with the use of Weber’s Chromotrope, showing the typical characteristics of microsporidia (Table 1). These spores measured 1 to 2 µm and were ovoid in shape with a clear vacuole-like polar zone. From the 45 stool samples microsporidia positive by stain, 37 (52.2%) were obtained from patients with diarrhea and 8 (6.3%) from non-diarrheic samples (Table 1). The presence of microsporidia spores in fecal samples from patients with diarrhea was compared with that from patients with no diarrhea, revealing a clear association between microsporidia and diarrhea with O.R.  =  18.2 (χ^2^ =  58.44; *p*< 10^–6^).

**Table 1:** Fecal samples for Microsporidia and *Cryptosporidium sp* by staining methods.

Diarrhea		Microsporidia		*Cryptosporidium* sp	
	Total (%)	Positive (%)	Negative (%)	Positive (%)	Negative (%)
**Yes**	67 (34.7)	37 (52.2)		11 (29.7)	26 (70.3)
			30 (47.8)	21 (70)	9 (30)
**No**	126 (65.3)	8 (6.3)		3 (37.5)	5 (62.5)
			118 (93.7)	9 (7.6)	109 (92.4)
**Total (%)**	193 (100)	45 (23.3)	148 (76.7)	44 (22.7)	149 (77.3)

Structures morfologically compatible with *Cryptosporidium* sp were detected in 44 of the HIV patients (22.7%) using Kinyoun stain. Thirty-two of positive patients presented diarrhea and 12 were found in non-diarrheic samples, supporting the well-known influence of *Cryptosporidium sp* on diarrhea (χ^2^ = 36,34; *p*< 10^–6^ ; OR = 8,7) (Table 1). Mixed infection was recorded between microsporidia and *Cryptosporidium sp* in 14 patients (31.8% of positives) (Table 1). In patients with diarrhea, the two types of microorganisms could be considered antagonistic as mixed-infections were lower than expected (χ^2^ = 10.77; *p*< 0.01) (Table 1).

### IFAT

The study by IFAT of microsporidia positive samples showed 8 positive samples. Five samples exhibited bright fluorescence when *E. bieneusi* monoclonal antibody was used; two reacted with *E. intestinalis* polyclonal antibody and one with *E. cuniculi* polyclonal antibody. Spores which reacted with the anti-*E. cuniculi* and anti-*E. intestinalis* polyclonal antibodies were observed in non-diarrheic samples, and *E. bieneusi* spores were identified in 1 non-diarrheic and 4 diarrheic samples by the monoclonal antibody used (Table 2).

**Table 2:** Microsporidian species identified by IFAT and PCR in fecal samples.

Diarrhea	IFAT		PCR	
	Species	n	Species	n
**Yes**	*E. bieneusi*	4	*E. bieneusi*	4
**No**	*E. bieneusi*	1	*E. bieneusi*	1
	*E. intestinalis*	2	*E. intestinalis*	2
	*E. cuniculi*	1		

n =  number of samples.

### PCR

A total of 7 cases, five for *E. bieneusi* and 2 for *E. intestinalis* were confirmed by amplification of DNA isolated from positive samples in the staining technique with specific primers for the most common microsporidia infecting humans (Table 2).

### DNA Sequencing Analysis

Three PCR positive samples for *E. bieneusi* yielded enough PCR products for partial sequencing of the ITS region of the rDNA genes. The Nigerian genotypes reported here are included in Group I. For comparison with 172 sequences from the GenBank, the gaps were excluded, and 139 sites were available for analysis. Fifty sites were polymorphic and generated 60 different haplotypes. One of the sequences obtained from the Nigerian isolates showed the same haplotype as genotype B [30]. Since we were unable to analyze the entire ITS, the sequences of the other two isolates coincided with several similar genotypes: P, type IV, UG2145, Peru3, PtEb IV or PtEbV [63]. The diversity in the ITS of *E. bieneusi* is very high but, within Group I, the differences between any pair of sequences involve only a small number of mutational events.

**Figure 1:**
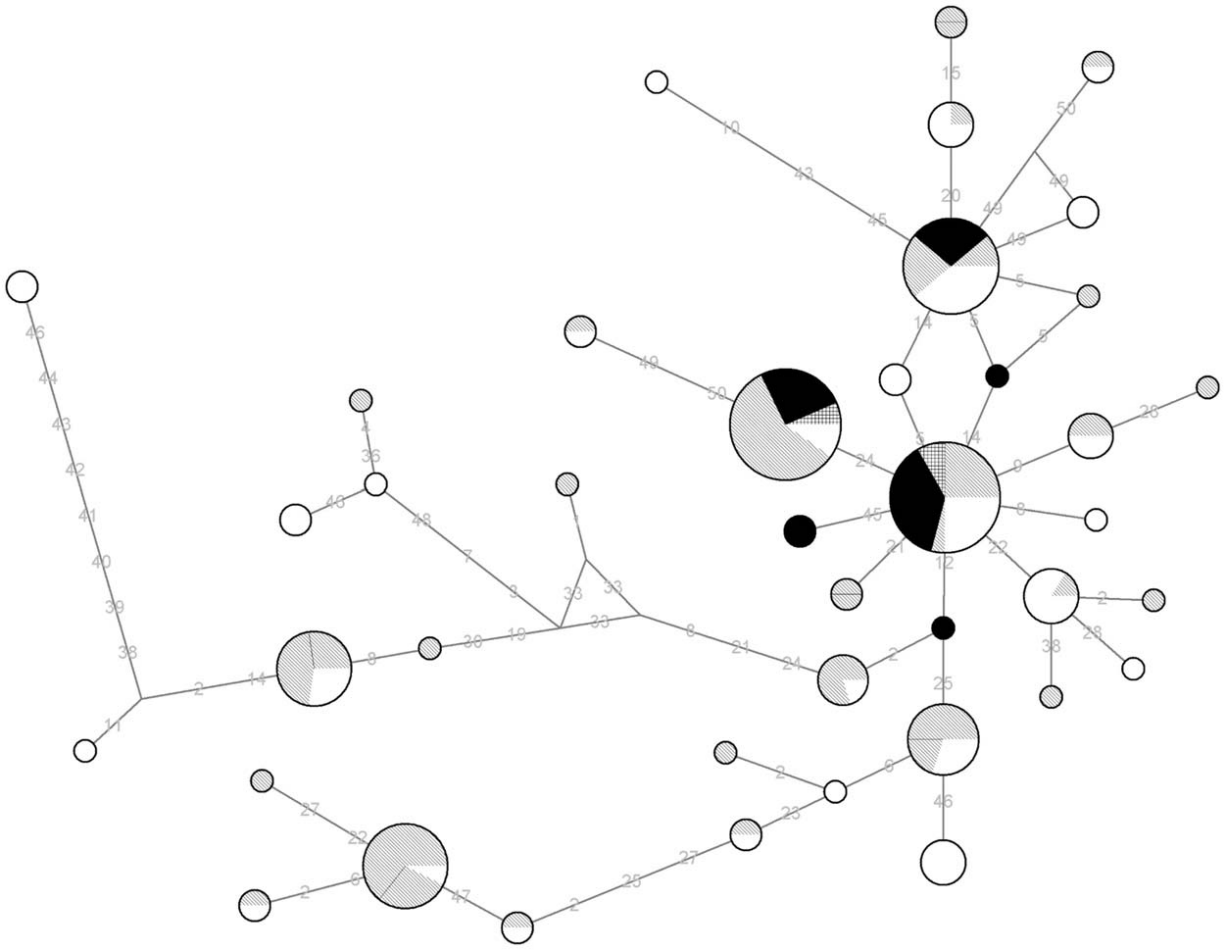
Median-joining network for the different haplotypes. Circles are proportional to the frequency of each haplotype. The colour of each sector refers to the host species: Nigerian samples (squared sectors), other African haplotypes (black), Europe and Asia (grey lines), North and South America (white). The lengths of the segments are proportional to the number of mutations involved between two haplotypes.

In order to locate the Nigerian samples within the wide variability of *E. bieneusi,* Median Joining networks were constructed with the program NETWORK [61]. The network was simplified, reducing star-like clusters to the ancestral haplotypes with a single run of star contraction [62]. The network with the resulting 40 taxa is shown in Figure 1. Nigerian haplotypes, together with the other African isolates previously analyzed, occupy a central position in the network.

## Discussion

Diarrhea caused by opportunistic agents such as microsporidia, *Cryptosporidium* species or *Isospora belli* contributes to morbidity and mortality in AIDS patients. In developing countries, these infections taken together may likely account for a great number of cases of diarrhea in HIV/AIDS patients [64]. To date there is only two reports included in PubMed of microsporidia infection in patients from Nigeria. In the study carried out in a rural village (Magama Gumau) and in a township in north central Nigeria a prevalence of 39.6% and 47.3% in rural and urban dwellers was reported [41]. However, the stain method used to detect microsporidia was not Webeŕs reference method or Gram-chromotrope stain and no characterization of the microsporidia species was made. More recently, a study was carried out in children attending an outpatient department of a hospital in Oyo state, southwestern Nigeria [46]. Using PCR, 9.3% of the children were identified as positive for *E.bieneusi.* Therefore, to contribute to a more complete knowledge of microsporidia epidemiology in Nigeria, this study has evaluated and characterized the presence of microsporidia in 193 fecal samples from HIV-positive patients with and without diarrhea (67 and 126 samples respectively); showing 45 positive samples (23.3%) when Webeŕs stain was used. Most of the positive samples were identified in diarrheic stools (52.2%) and only 8 in non-diarrheic stools (6.3%), showing a significant association between microsporidia and diarrhea in the population studied (OR  =  18.2) such as had previously been reported in other countries [13], [18], [19], [22], [65]. Our results are in accordance with previous studies on the prevalence of microsporidia in HIV-positive patients, which ranges from 5 to 50% depending on the geographical location and the diagnostic technique employed [66]. This suggests that microsporidia are becoming highly prevalent intestinal parasites in developing countries such as Nigeria and thus the need to consider them as neglected etiological agents [16]. In reference to the African continent, our results are also in accordance with previous studies in HIV-positive patients with chronic diarrhea [67], [68].

To determine the species of microsporidia implicated in the study, two additional methods were used: IFAT and PCR (the gold standard). The use of IFAT allowed the identification of microsporidia species in only 8 fecal samples (Table 2).We detected a low positive result by IFAT compared with Webeŕs staining. However, the scarce parasitic load and the low specificity of Weber’s staining could be related with the differences observed. However, we can not discard the possibility that other microsporidia species were present in our samples.

In our study most of the positive samples by IFAT were *E. bieneusi* (4) followed by *E. intestinalis* (2).These results are supported by the fact that *E. bieneusi* and *E. intestinalis* are the most prevalent intestinal microsporida previously described in other countries including Africa [2], [40], [42], [46], [67], [72], [73], [74], [75], and *E. cuniculi* has only occasionally been found in the intestine of HIV-positive patients [12].

The PCR method identified 7 positive samples belonging to the species *E. bieneusi* and *E. intestinalis* (Table 2). The PCR technique is believed to be the most sensitive method for species identification, but it is expensive and not affordable in many clinical diagnostic laboratories. In the case of stool samples, the main problem is the appearance of false-negative results due to a low parasite-DNA concentration, and the presence of PCR inhibitors [13], [69], [70]. Following from this, we should point out that we found a high presence of PCR inhibitors that had not been eliminated by the usual common methods [53], [55]. This fact is supported by the number of control samples spiked with the cloned microsporidia DNA which were impossible to amplify. Their proportion was higher than those usually found in previous studies with fecal samples from other countries [49], [71]. We can only speculate that the characteristics of the diets or treatments followed by these patients may contain PCR inhibitors. In addition to the above difficulties, it is also necessary to consider that human samples positive by the staining methods may not necessarily amplify with the specific primers used, due to other causes such as the possible presence of microsporidia other than the species studied, the overload of extruded spores in samples containing a low parasitic load, etc. The positive PCR results (3.6%) are much lower than those previously obtained, also by PCR, in Zimbabwean HIV-positive patients (51%) [67]. However, in a study carried out in Mali, a country nearer Nigeria, *E. bieneusi* was found in 6.8% of HIV-positive patients and in 9% of HIV-negative individuals [72], and these data also correlates with the 9.3% of children recently identified in Nigeria as positive for *E. bieneusi* [46].

Few studies on microsporidia exist in Africa, but previous reports identified *E. bieneusi* as the most prevalent microsporidia infecting humans [40], [42], [46], [67], [72], [73], [74], [75]. In the present report, *E. bieneusi* is described for the first time as the most prevalent microsporidia in HIV-positive patients with diarrhea in Nigeria. Additionally, *E. intestinalis* was also detected for the first time in this country although a previous report [41] described the presence of *E. bieneusi*/*E. intestinalis* but a without specific differentiation been performed. It is interesting to note the detection of *E. cuniculi* by IFAT in a fecal sample, although there are no previous references of this microsporidia in humans from the African continent. It is also noteworthy that in a study carried out in Nigeria in domestic rabbits, an *E. cuniculi* seropositivity of 16.5% was described, suggesting the zoonotic origin of this infection [76].

A high intraspecific variability in *E. bieneusi* has been described by several authors based on ITS of the rRNA gene. Most sequences found up to now for the ITS of *E. bieneusi* belong to Group I, including those found in our samples, and only a minority to divergent Groups II, III and IV [59]. Recently, Breton et al., [43] reported the prevalence in Cameroon and Gabon of several genotypes (A, B, D, K) included in Group I of the most common *E. bieneusi* haplotypes, and a divergent genotype CAF4 (Group II) whose significance is not yet clear [59]. It has been suggested that some genotypes of *E. bieneusi* could possibly be host adapted genotypes and may be of importance to public health with reference to specific animal groups [77]. However, a recent phylogenetic analysis showed that humans may become infected by *E. bieneusi* from different sources, including human-to-human, but there is also a certain degree of host-specificity [59]. The sequences of the three Nigerian samples were similar to previously described genotypes found in *E. bieneusi* isolates from Africa, Europe, Asia and North and South America, and also from a number of different host species, including humans, cattle, pets and wild animals [30], [59], [78]. According to their position in the network (Figure 1) they presumably represent the most ancient haplotypes of *E. bieneusi*. It is interesting to note that such central locations in the network apply to all the haplotypes found so far in Africa, including those recently reported by Ayinmode et al. [46]. This result supports the idea that genotypes of *E.bieneusi* found in HIV-positive patients are closely related to those from several animals, thereby supporting the possibility of zoonotic transmission of the strains.

Finally, the prevalence of *Cryptosporidium* was established in 22.7% of the 193 fecal samples studied, with 47.7% and 9.5% in diarrheic and non-diarrheic samples respectively. These data are similar to those found for microsporidia using Trichrome stain (23.3% total, and 52.2% and 6.3% in diarrheic and non-diarrheic samples respectively), and are in accordance with previous reports indicating that *Cryptosporidium* infection is not the primary cause of diarrhea in HIV-positive patients in Africa [40], [67], [72]. Moreover, microsporidia and *Cryptosporidium* were found coinfecting 30% of *Cryptosporidium* positive patients in this study, although this was not surprising as it is consistent with previous observations of both parasites in HIV/AIDS patients [65], [67], [79], [80], [81].

In conclusion, our results provide new data on microsporidia epidemiology on the African continent, where the control of HIV/AIDS is already due to limited access to HAART. Because of this, the presence of chronic intestinal microsporidiosis and cryptosporidiosis should be suspected in HIV/AIDS infected patients with persistent diarrhea, malabsorption and progressive weight loss. More importantly, there is further need to investigate other clinical and environmental samples in order to identify the major sources of these pathogens in human, in the environment.
